# The acceptability and feasibility of an internet-administered, guided, low-intensity cognitive behavioural therapy intervention for parents of children treated for cancer: findings from a qualitative study involving public contributors

**DOI:** 10.1186/s12888-025-06897-y

**Published:** 2025-05-16

**Authors:** Ella Thiblin, Johan Lundgren, Mattias Bergqvist, Tho Huynh, Christina Reuther, Sandra Rösler, Joanne Woodford, Louise von Essen

**Affiliations:** 1 CIRCLE– Complex Intervention Research in Health and Care, Department of Women’s and Children’s Health, Uppsala University, Uppsala, Sweden; 2 Division of Nursing Sciences and Reproductive Health, Department of Health, Medicine and Caring Sciences, Linköping University, Norrköping, Sweden; 3 ENGAGE Study Public Contribution Group, Uppsala, Sweden

**Keywords:** Childhood cancer, Parents, Internet-administered interventions, Low-intensity CBT, Public contribution in research

## Abstract

**Background:**

Despite parents of children treated for cancer commonly reporting psychological difficulties such as symptoms of depression and anxiety, there is a lack of evidence-based psychological interventions tailored to their needs. We therefore developed an internet-administered, guided, low-intensity cognitive behavioural therapy-based self-help intervention (EJDeR). We examined the acceptability and feasibility of the intervention and study procedures in the single-arm feasibility trial ENGAGE. Results suggested the intervention and study procedures are feasible and acceptable. However, a need for modifications and refinements to the intervention and study procedures was identified. We conducted a qualitative interview study to explore the acceptability and feasibility of the intervention and study procedures from the perspective of parents to inform modifications and refinements to the intervention and study procedures.

**Methods:**

Semi-structured interviews were conducted with 52 parents (17 fathers, 35 mothers). A public contribution group (three parents of children treated for cancer) analysed the data independently from the research team and contributed to dissemination. An inductive content analysis was performed using the Framework Method.

**Results:**

The intervention was perceived as acceptable and relevant, and parents expressed positive attitudes toward internet-administered support. However, parents identified a need for adaptations to intervention content, e.g., a greater focus on the family, trauma, and the cancer experience. Barriers to engagement were also identified, including technical difficulties and a need for modifications to improve intervention user-friendliness and smartphone and/or tablet compatibility. Study procedures were also perceived as acceptable and feasible. However, a need was identified to improve parents’ understanding of the study and to reduce the amount and frequency of assessments. The public contributors’ analysis was similar to that of the research team. However, the research team overlooked important nuances. For example, the public contributors’ analysis highlighted parents’ difficulties distinguishing between the intervention and study procedures.

**Conclusions:**

The intervention and study procedures were perceived as acceptable and feasible. However, the need for important modifications and refinements were suggested to improve future acceptability and feasibility. Involving public contributors in the analysis resulted in developing a more comprehensive and nuanced understanding of the data.

**Trial registration:**

ISRCTN 57,233,429 (Registration date: 19/04/2018); ISRCTN 18,404,129 (Registration date: 25/11/2019).

**Supplementary Information:**

The online version contains supplementary material available at 10.1186/s12888-025-06897-y.

## Background

Each year around 400,000 children aged 0–19 years are diagnosed with cancer worldwide [1]. As cancer treatments advance, survival rates are increasing [2], and in Northern Europe, the five-year survival rate has exceeded 80% [3]. The end of cancer treatment is a period of significant psychological vulnerability for parents, with some reporting psychological difficulties, such as symptoms of depression, anxiety, and post-traumatic stress symptoms (PTSS) [4–9]. Despite this, there is a lack of evidence-based psychological interventions tailored to parents’ needs [10].

Over the past two decades, the use of low-intensity cognitive behavioural therapy (LICBT) as a way to improve access to psychological support for common psychological difficulties such as depression and anxiety has increased worldwide [11]. LICBT techniques are delivered via self-help interventions (e.g., in audio, digital format, and print format [11]), including internet-administered cognitive behavioural therapy (iCBT) [12]. Whilst unguided iCBT self-help interventions are effective, guided self-help interventions (i.e., supported by a trained professional) are associated with higher effect sizes than unguided iCBT for both depression [13] and anxiety disorders [14]. iCBT may also address barriers to seeking support, such as guilt, lack of time, and geographical location [15] via increased anonymity, privacy, and flexibility of delivery [16].

An internet-administered, guided, LICBT-based self-help intervention, EJDeR [Swedish acronym for int**E**rnetbaserat s**J**älvhjälpsprogram för föräl**D**rar till barn som avslutat en behandling mot canc**eR**], may address the unmet support needs of parents of children treated for cancer [17]. We developed EJDeR by undertaking iterative development phase research following the Medical Research Council (MRC) complex intervention framework [18]. Intervention development was informed by a systematic review [5], semi-structured interview studies [19, 20], a single-arm trial and conceptualisation of distress [21], participatory action research [22], and a cross-sectional web-based survey [23]. Public contribution in research [24] was used to enhance intervention acceptability and relevancy [17]. EJDeR targets symptoms of depression and generalised anxiety disorder (GAD), common psychological difficulties reported by parents of children treated for cancer [4, 9]. EJDeR is delivered on the U-CARE Portal (Portal) [25] and includes four modules: (1) introduction and psychoeducation (IPE); (2) behavioural activation (BA) for depression; (3) worry management (WM) for GAD; and (4) relapse prevention (RP).

Next, by conducting the single-arm feasibility trial ENGAGE [26, 27], following the feasibility phase of the MRC complex interventions framework [18], we examined methodological (e.g., sample size, study design), procedural (e.g., recruitment and retention), and clinical uncertainties (e.g., intervention acceptability) before progression to an external pilot randomised controlled trial (RCT) (the CHANGE-pilot trial) to prepare for the design and conduct of a future superiority RCT of EJDeR plus usual care (UC) versus UC [26]. Semi-structured interviews at baseline (before accessing EJDeR) explored parents’ self-reported concerns [28] and coping strategies used to cope with cancer-related distress and concerns [29]. Post-treatment interviews (12 weeks after accessing EJDeR) explored the acceptability and feasibility of EJDeR and ENGAGE study procedures. Public contribution in research was embedded during different stages of ENGAGE, including the analysis of post-treatment interviews.

Results from ENGAGE demonstrated the overall acceptability and feasibility of EJDeR and ENGAGE study procedures [26], warranting progression to the CHANGE-pilot trial. However, not all progression criteria [30] were met, suggesting a need for some modifications and refinements to EJDeR and refinements to study procedures prior to progressing to the CHANGE-pilot trial. Regarding EJDeR, the overall adherence rate was 47.9%, below the 50.0% progression criteria. Adherence rates to the minimum treatment dose (defined as attending an initial assessment session with an e-therapist, completing the IPE module, completing the BA or WM module, and attending a mid-intervention booster session with an e-therapist) [26] varied by the first LICBT module opened by parents: 76.9% for BA and 50.0% for WM. For ENGAGE study procedures, Portal assessment completion was 65.6% at post-treatment (12 weeks) and 67.8% at follow-up (6 months), both slightly below the 70.0% progression criteria. Weekly Portal assessment completion rates during the intervention also failed to meet the 70.0% progression criteria, decreasing from 65.7% in week 1 to 38.9% in week 11.

Post-treatment interviews exploring the acceptability and feasibility of EJDeR and ENGAGE study procedures may help inform future modifications and refinements to the intervention and study procedures [31]. Exploring intervention acceptability from the perspective of parents (i.e., intervention relevancy, usefulness, and barriers and facilitators to working with the intervention) may inform clinical and technical modifications and refinements to EJDeR to improve future intervention adherence. Further, given low Portal assessment completion rates threaten the reliability, validity and usability of trial results [32], it is important to understand the acceptability and feasibility of study procedures (e.g., potential reasons for low Portal assessment completion rates) to inform further modifications and refinements to study procedures in the future CHANGE-pilot trial.

### Aim and objectives

The present study aimed to explore the acceptability and feasibility of EJDeR and ENGAGE study procedures from the perspective of parents to inform future modifications and refinements to the intervention and study procedures before progression to the CHANGE-pilot trial. Specific objectives were to explore parents’ (1) overall impressions of EJDeR; (2) barriers to engagement with EJDeR; (3) reasons for poor adherence; (4) suggestions for adaptations to EJDeR; (5) use of EJDeR exercises in everyday life; (6) perceptions of EJDeR’s impact on mood and everyday life; and (7) perceptions of ENGAGE study procedures.

## Methods

We conducted a qualitative interview study embedded in the ENGAGE feasibility trial, with data collected via post-treatment (12 weeks) interviews with parents who participated in ENGAGE. To improve research quality by incorporating diverse perspectives into data analysis and interpretation [33], and to facilitate meaningful and collaborative public contribution [34], a public contribution group [24] of three public contributors (parents of children treated for cancer) analysed the data independently from the research team and contributed to dissemination [34, 35]. Methods and findings are reported following the consolidated criteria for reporting qualitative research (COREQ) (Additional file 1) [36].

### Theoretical framework

An inductive content analysis was conducted using the Framework Method [37, 38]. A pragmatic research paradigm was adopted, selecting methods suited to the research aim with findings considered transferable to other similar contexts [39]. An inductive approach was chosen based on the assumption that pre-existing knowledge of theoretical frameworks that could have informed a deductive approach, e.g., the Theoretical Framework of Acceptability [40], would vary between public contributors and research team members. Consequently, public contributors would have needed additional training, which may have resulted in unintended harms e.g., added burden on public contributors and increased power imbalances between the public contributors and the research team [41].

### Setting

#### The ENGAGE feasibility trial

The published study protocol [27] and main results paper [26] provide full details concerning the design of ENGAGE. Recruitment took place between July 2020 and November 2020 through postal study invitations and online advertisements. Eligible participants were parents of children (0–18 years at cancer diagnosis) who completed treatment 3 months to 5 years previously; residing in Sweden; able to access the internet, a mobile phone, and Bank-ID (a Swedish citizen digital identification system); having a self-reported need for psychological support related to the child’s cancer; and able to read and write Swedish. Exclusion criteria included self-reported or clinician assessed severe and enduring psychological disorder; substance misuse; current psychotherapy attendance; and acute suicidality.

Parents provided consent via the Portal, a web-based platform, designed to deliver internet-administered psychological support and support digital research activities (e.g., web-based consent and data collection) [25]. Eligibility was confirmed via a telephone interview, including parts of the Mini-International Neuropsychiatric Interview (M.I.N.I.) version 7.0.0 [42], conducted by a licensed psychologist. Eligible parents were invited to an optional semi-structured telephone interview with a licensed psychologist to explore self-reported concerns [28] and coping strategies used to cope with cancer-related distress and concerns [29]. Subsequently, after completing the Portal assessment at baseline (psychological and health-economic measures comprising 11 questionnaires), parents gained access to EJDeR and were allocated an e-therapist (referred to as *parent guide* to parents). Portal assessments were also collected at post-treatment (12 weeks) and follow-up (6 months). Reminders were sent (via SMS, e-mail or telephone, according to preference) if Portal assessments were not completed within one week. If Portal assessments remained uncompleted after two weeks, parents were contacted and offered the option of completing the Portal assessment over the telephone. Weekly Portal assessments (5 questionnaires) were collected during the 12-week intervention period.

#### The EJDeR intervention

EJDeR was delivered on the Portal and could be accessed via computers, smartphones, and tablets. The clinical protocol has been published [17] following the Template for Intervention Description and Replication (TIDieR) checklist [43].

The intervention was delivered over 12 weeks and consisted of four modules: IPE, BA, WM, and RP. BA is a LICBT technique for depression [44], and WM, including two techniques, Worry Time and Problem Solving, is a LICBT technique for GAD [44]. Modules included text, illustrations, audio and video files, case vignettes, in-module online exercises, and weekly online homework exercises. EJDeR was guided by e-therapists with education in CBT and LICBT. Ten e-therapists were final-year psychology program students, two were licensed clinical psychologists, and one was a CBT therapist. E-therapists held an initial assessment session with parents (telephone or video-conferencing) shortly after accessing EJDeR. During the initial assessment session, a collaborative decision to work with BA or WM was made, depending on the parent’s main difficulty. E-therapists provided weekly written feedback via the Portal and a mid-intervention booster session (telephone or video-conferencing). After completing BA or WM, parents could access the remaining LICBT module. All parents received the RP module at the end of EJDeR.

### Recruitment and sample

In total, 81 parents consented to participate in ENGAGE. During eligibility interviews, one was excluded (acutely suicidal *n*= 1), and eight dropped out from the study before accessing EJDeR (no reasons provided). In total, 72 gained access to EJDeR. During the intervention, one parent was excluded (severe and enduring mental health difficulty *n* = 1), and seven dropped out from the study (no need for support *n* = 1, too stressful *n* = 1, no reason provided *n* = 5). At post-treatment (12 weeks), all parents remaining in the trial (*n* = 64) were invited to take part in the semi-structured interview, regardless of whether they adhered to EJDeR or not. Of the invited parents, three (2 fathers, 1 mother) declined participation in the interview (no need for support *n* = 2, too stressful *n* = 1), and eight (2 fathers, 6 mothers) were lost to follow-up, resulting in 53 parents participating in the interviews. One interview was excluded from the analysis due to its short length and lack of information. Data is reported for 52 parents (17 fathers, 35 mothers).

### Sample description

Baseline sociodemographic and clinical characteristics are reported in Additional File 2. Parents had a mean age of 41.7 years, 32.7% were fathers, most had tertiary level education, were house owners, were born in Sweden, and cohabited with a partner. At baseline, parents’ mean depression score (Patient Health Questionnaire 9-item scale [PHQ-9]) [45] was 6.3 (SD 4.6), and their mean anxiety score (Generalised Anxiety Disorder 7-item scale [GAD-7] [46] was 5.9 (SD 4.1). Parents mean PTSS score (Post-traumatic Stress Disorder Checklist for DSM-5 [PCL-5]) [47] was 15.5 (SD 10.0), and using the adapted version of the Post-traumatic Stress Disorder Checklist-Civilian version [PCL-C]) [48] was 31.0 (SD 8.4). In total, 63.5% (33/52) of parents interviewed adhered to EJDeR (12/17; 70.6% fathers, 21/35; 60.0% mothers), a higher percentage compared to the overall ENGAGE sample, where 47.9% (34/71) adhered to EJDeR (12/25; 48.0% fathers, 22/46; 47.8% mothers). Selected sample characteristics are reported in Table 1.

**Table 1:** Sample characteristics (*n* = 52)

Parent ID	Overall adherence	LICBT module(s) opened	Relationship to the child	Age (years)	Depression severity at baseline^a^	Anxiety severity at baseline ^b^
Parent 1	Adherer	BA, WM	Father	30–39	Minimal	Minimal
Parent 2	Adherer	BA, WM	Father	40–49	Minimal	Minimal
Parent 3	Adherer	BA, WM	Father	30–39	Mild	Minimal
Parent 4	Adherer	BA, WM	Father	40–49	Mild	Minimal
Parent 5	Adherer	BA, WM	Mother	30–39	Minimal	Minimal
Parent 6	Adherer	BA, WM	Mother	30–39	Minimal	Minimal
Parent 7	Adherer	BA	Father	40–49	Minimal	Minimal
Parent 8	Adherer	BA	Father	40–49	Minimal	Minimal
Parent 9	Adherer	BA	Father	40–49	Minimal	Minimal
Parent 10	Adherer	BA	Father	40–49	Moderate	Moderate
Parent 11	Adherer	BA	Mother	30–39	Minimal	Mild
Parent 12	Adherer	BA	Mother	30–39	Mild	Mild
Parent 13	Adherer	BA	Mother	30–39	Mild	Mild
Parent 14	Adherer	BA	Mother	30–39	Mild	Mild
Parent 15	Adherer	BA	Mother	30–39	Mild	Mild
Parent 16	Adherer	BA	Mother	40–49	Mild	Mild
Parent 17	Adherer	BA	Mother	40–49	Mild	Mild
Parent 18	Adherer	BA	Mother	40–49	Mild	Moderate
Parent 19	Adherer	BA	Mother	30–39	Moderate	Mild
Parent 20	Adherer	BA	Mother	40–49	Moderate	Moderate
Parent 21	Adherer	BA	Mother	40–49	Moderate	Moderate
Parent 22	Adherer	BA	Mother	40–49	Severe	Severe
Parent 23	Adherer	WM	Father	40–49	Minimal	Minimal
Parent 24	Adherer	WM	Father	40–49	Minimal	Mild
Parent 25	Adherer	WM	Father	40–49	Mild	Minimal
Parent 26	Adherer	WM	Father	30–39	Mild	Mild
Parent 27	Adherer	WM	Mother	50–59	Minimal	Minimal
Parent 28	Adherer	WM	Mother	30–39	Minimal	Mild
Parent 29	Adherer	WM	Mother	50–59	Mild	Minimal
Parent 30	Adherer	WM	Mother	40–49	Mild	Mild
Parent 31	Adherer	WM	Mother	40–49	Mild	Mild
Parent 32	Adherer	WM	Mother	40–49	Mild	Moderate
Parent 33	Adherer	WM	Mother	40–49	Mild	Moderate
Parent 34	Non-adherer	BA	Mother	30–39	Minimal	Minimal
Parent 35	Non-adherer	BA	Mother	40–49	Mild	Mild
Parent 36	Non-adherer	BA	Mother	40–49	Moderate	Severe
Parent 37	Non-adherer	BA	Mother	40–49	Moderately severe	Moderate
Parent 38	Non-adherer	WM	Father	40–49	Minimal	Minimal
Parent 39	Non-adherer	WM	Father	40–49	Minimal	Minimal
Parent 40	Non-adherer	WM	Father	40–49	Mild	Minimal
Parent 41	Non-adherer	WM	Father	30–39	Mild	Mild
Parent 42	Non-adherer	WM	Father	50–59	Mild	Mild
Parent 43	Non-adherer	WM	Mother	40–49	Minimal	Minimal
Parent 44	Non-adherer	WM	Mother	30–39	Mild	Mild
Parent 45	Non-adherer	WM	Mother	30–39	Moderate	Moderate
Parent 46	Non-adherer	None	Mother	30–39	Minimal	Minimal
Parent 47	Non-adherer	None	Mother	30–39	Minimal	Minimal
Parent 48	Non-adherer	None	Mother	40–49	Minimal	Mild
Parent 49	Non-adherer	None	Mother	40–49	Mild	Minimal
Parent 50	Non-adherer	None	Mother	30–39	Mild	Mild
Parent 51	Non-adherer	None	Mother	30–39	Mild	Mild
Parent 52	Non-adherer	None	Mother	50–59	Moderate	Mild

^a^Depression severity cutoffs: 0–4 = Minimal; 5–9 = Mild; 10–14 = Moderate; 15–19 = Moderately severe; 20–27 = Severe [44]

^b^GAD severity cutoffs: 0–4 = Minimal; 5–9 = Mild; 10–14 = Moderate; 15–21 = Severe [45]

### Research team, public contribution group, and reflexivity

Interviews were conducted within 28 days of ending EJDeR by four licensed psychologists external to the research team, two licensed psychologists (including ET), and one CBT therapist internal to the research team. Interviewers (female *n* = 6, male, *n* = 1) had 3–30 years of clinical experience. Three interviewers were also e-therapists, but they did not interview parents they guided. Two of the seven interviewers had prior experience in conducting qualitative interviews.

Data analysis by public contributors was conducted by MB and TH, fathers of a child previously treated for cancer, and SR, a mother of a child previously treated for cancer. MB, TH, and SR also participated in ENGAGE as study participants and were recruited from a list of ENGAGE participants who had expressed an interest in collaboration with the research team when asked via e-mail at the end of ENGAGE. No specific inclusion criteria were applied for the public contribution group, and public contributors had no experience in conducting qualitative research.

Data analysis by research team members was conducted by JL, a male researcher (PhD Nursing Sciences); CR, a female PhD student (MSc Public Health); and ET, a female PhD student and licensed psychologist who also worked as an e-therapist (MSc Psychology). All research team members had prior experience in qualitative research. LvE, a female professor in Healthcare Sciences and principal investigator for ENGAGE, and JW, a female assistant professor in Healthcare Sciences (PhD in Psychology), provided peer examination. LvE and JW are experienced in conducting, teaching, and supervising qualitative research and co-authored the EJDeR intervention.

### Data collection

Interviews were conducted between September 2020 and April 2021, lasting between 15 and 46 min (mean 29 min, SD 8.0). Following a semi-structured interview guide (Additional File 3), interviews explored parents’ views concerning (1) overall expectations of EJDeR and ENGAGE; (2) EJDeR exercises and use of exercises in everyday life; (3) guidance from the e-therapist; (4) the Portal; (5) study questionnaires and reminders; (6) reasons for poor adherence to EJDeR; (7) suggestions for improving EJDeR; and (8) advantages and disadvantages of internet-administered support compared to face-to-face treatment. Interviewers were instructed to encourage parents to answer in their own words at their own pace, and to explore responses by asking follow-up questions. Data collection was not guided by data saturation but predetermined by the number of parents reaching post-treatment (12 weeks) and agreeing to participate. Interviews were audio-recorded on an Olympus Digital Voice Recorder WS-853 and transcribed verbatim in Swedish by a professional transcriber, with identifying information (e.g., names) removed.

### Data analysis

Interviews were analysed using the Framework Method [37, 38], with a low level of interpretation. Two separate data analyses were conducted, one by the public contribution group (MB, TH, SR) and one by research team members (JL, CR, ET). Research team members conducted their analysis before public contributors to minimise the influence of the public contributors’ analysis on the research teams’ analytical decision-making.

Initially, four public contributors were involved in the analysis, dividing 48 interview transcripts between them (i.e., they did not analyse their own or each other’s interviews). However, one public contributor withdrew from the group during the initial coding phase, and transcripts assigned to this person were removed from the public contributors’ analysis to reduce burden on the remaining public contributors. In total, the public contribution group analysed 36/52 interview transcripts. Potentially identifying information (e.g., city, hospitals visited) was removed from transcripts analysed by public contributors. Research team members divided all 52 interview transcripts between them. ET did not analyse interviews she conducted or those with parents for whom she was the e-therapist.

JL, CR, and ET trained public contributors in the Framework Method [37, 38] in a series of three face-to-face workshops and offered online supervision (via Zoom) on demand. During training workshops and supervision, research team members provided support to public contributors but placed active emphasis on minimising the impact of their own data interpretation on the public contributors’ analytical decision-making. A separate methods article will be published, providing a detailed outline of, alongside reflections on, the process of involving public contributors in data analysis.

Analysis was carried out by both public contributors and research team members following five steps: (1) *Familiarisation*: Interview transcripts were read multiple times; (2) *Developing an initial coding framework*: Interview transcripts were independently inductively coded in two cycles, one initial and one where codes were refined and reduced. Public contributors coded by hand on printed transcripts, and research team members used Nvivo version 1.7.1. Next, separate coding workshops were held with public contributors and research team members, respectively. During workshops sets of codes were collaboratively agreed upon and refined through discussions, e.g., merging similar codes into one code and renaming codes found to be too detailed or abstract. Codes were then collaboratively grouped into categories. Research team members facilitated workshops for public contributors; (3) *Indexing*: Interview transcripts were indexed according to the initial frameworks, applying codes and categories identified in step four to the transcripts. Regular meetings were held with public contributors and research team members, respectively, where frameworks were discussed and amended. Research team members were present at meetings held with public contributors. Public contributors indexed by hand on printed transcripts and research team members used Nvivo. Salient quotations were marked and thereafter discussed and agreed upon collaboratively within each group; (4) *Charting*: Indexed data were charted into matrices in Microsoft Excel. One matrix was developed for each category with relevant codes organised in columns, and data from each parent (i.e., data summaries and illustrative quotations) were organised in rows. See Table 2 for an example of a matrix for the research team’s category “Opinions on internet-administered treatments”. Charting for the public contributors was conducted by research team members (ET and CR) to facilitate and reduce burden given public contributors were not using Nvivo; (5) *Mapping and interpretation*: Separate mapping and interpretation workshops were held with public contributors and research team members respectively, with research team members facilitating the public contributors’ workshops. During mapping and interpretation workshops, category content was described, patterns within and between cases searched for, and interpretations discussed. Findings were summarised in the form of written memos. The research team led report writing, including organising categories into key areas.

**Table 2:** A worked example of charted data for the category “opinions on internet-administered treatments”. Cases are listed in the rows, and data under each code in the columns

Parent ID	Codes				
	Internet-administered treatments - barriers	Internet-administered treatments - flexibility	Internet-administered treatments - demands on user	Internet-administered treatments - relational aspects	Internet-administered treatments - accessibility
Parent 42			I spoke to [parent guide] a little about motivation and things like that, and I suppose I may not be alone in quitting somewhere in the middle [of the intervention] or before it’s finished. That’s the downside, it’s kind of like when you work out at a gym. It’s better to have a personal trainer because you need to be there at a certain time, and you have someone who gives feedback on-site.	Interviewer: So, for the motivation part it can be an advantage to meet face to face. Parent: Yes, I think so.	
Parent 36	No, I can’t see any downsides				It’s digital and it’s convenient and it’s easy.
Parent 32		I can decide the time, that’s positive.			

### Trustworthiness

Several strategies were used to establish trustworthiness [49]. An audit trail was maintained by documenting the data analysis process and decisions. Memos were written by public contributors and research team members to facilitate drafting the final report and ensure the different interpretive perspectives within each team were not lost [50]. Peer examination involved discussing research procedures and findings with team members not involved in the analysis. Researcher triangulation was used by involving multiple researchers in coding, analysis, and interpretation. Supporting quotations alongside parent ID, selected characteristics (relationship to the child, adherer/non-adherer, LICBT module(s) opened), and ENGAGE feasibility trial setting, including a detailed description of EJDeR provides a comprehensive understanding of the context, strengthening credibility and facilitating transferability of findings.

## Results

Results are organised under four key areas: Expectations and needs; Barriers, facilitators, and experiences with participation; Intervention delivery and support; and Areas for improvements. Categories created by the public contributor group and the research team group are presented under these key areas. Key areas, categories, and which group created each category are presented in Table 3. In the results, e-therapists are referred to as parent guides, as that was the word used by parents.

**Table 3:** Overview of key areas and categories resulting from the research team and public contribution group analysis

Key area	Analysis team	Category
Expectations and needs	Public contributors	Expectations/information beforehand
	Research team	Before the study
		Need for support
Barriers, facilitators and experiences with participation	Public contributors	Barriers to participation
		General experiences with participation
		Reminders
		Positive experiences with participation
	Research team	Barriers to engagement
		Acceptability of study procedures
		Appreciated parts of the intervention
		Mechanisms of change and impact
Intervention delivery and support	Public contributors	Parent guide
	Research team	Opinions on internet-administered interventions
		Communication and support
Areas for improvement	Public contributors	Wishes, opinions, and suggestions for improvement
	Research team	Suggestions and improvements

### Expectations and needs

#### Expectations/information beforehand (public contributors)

By taking part in ENGAGE and EJDeR, parents expected to receive help to feel better, and to contribute to developing and improving the intervention:

“[My expectations were] *maybe to come to an understanding or the like […] You always strive to feel a little* [bit] *better than you do. And maybe that was it.*” (Participant 44, mother, non-adherer, WM).

For some parents, the primary motivation for participation was contributing to knowledge about parents’ experiences of being a parent to a child treated for cancer and helping others, rather than receiving support for their own needs:

“*Yes*,* because my reason for joining the intervention was that I wanted to help everyone who is in this situation*,* more than that I had a need that I wanted help with*.” (Parent 24, father, adherer, WM).

Parents with specific study and intervention expectations generally found these were met. They perceived the study information as accurate, however some mentioned not understanding the study duration.

#### Before the study (research team)

The study invitation letter was generally considered to include adequate information, although some parents reported not reading it carefully. Parents expressed a range of study and intervention expectations, such as gaining tools for self-understanding, talking to someone, receiving help with depression and anxiety, addressing the experience of being a parent of a child treated for cancer, and learning about the connection between behaviours and emotions. Other parents approached the study with no expectations, keeping an open mind:

“*Yeah*,* I didn’t have that many expectations. I thought I started it* [the study] *with an open mind really*,* so yeah*,* no*,* I didn’t have a lot of expectations.*” (Parent 6, mother, adherer, BA & WM).

However, some parents voiced participating in the study to help others or expected to be involved in intervention development as opposed to receiving an intervention. Others anticipated a more personalised intervention or more focus on the child and/or family and cancer experience:

“*I had an expectation that there would be more details about how you experienced things related to the child’s illness*.” (Parent 46, mother, non-adherer, no LICBT module opened).

#### Need for support (research team)

Parents described the support received positively, with some voicing encountering difficulties finding support elsewhere, having previously actively searched for support without success. The intervention was generally perceived as relevant, though some reported feeling “*too well*” to benefit:

“*I don’t really think I was in the target group*,* because I felt a bit too well*,* or feel a bit too well.*” (Parent 43, mother, non-adherer, WM).

Most parents would recommend the intervention to other parents in a similar situation. Generally, parents thought the intervention should be offered a few months to a year after cancer treatment ended, with no upper time limit for use:

“*Yes*,* not directly* [after], *I don’t think. Then of course it’s very individual… it may be individual but… I don’t know*,* a year maybe*,* or two* [on when the intervention should be offered].” (Parent 15, mother, adherer, BA).

However, parents who participated close to five years after end of treatment tended to say that receiving the intervention now was a bit too late.

### Barriers, facilitators and experiences with participation

#### Barriers to participation (public contributors)

Barriers to participation included parents’ own health issues, and underestimating the time needed for the intervention, with the intervention becoming an extra stress factor. Wrong timing, such as working with the intervention during the COVID-19 pandemic, was also reported. Difficulties understanding parts of the intervention, getting started with exercises, lack of personal contact with the parent guide, and feelings of loneliness were voiced as barriers. Further, parents reported that the amount and frequency of questionnaires hindered engagement, and were experienced as confusing, repetitive, time-consuming, and stressful:

“*The experience was stressful. I had scheduled in my calendar to receive reminders every Thursday to address these questions*,* but there never seemed to be a convenient time for it*,* so I ended up feeling rushed to answer them*.” (Parent 16, mother, adherer, BA).

Questions about suicide were specifically mentioned as eliciting negative reactions among parents and were perceived as being asked too frequently.

The Portal was described as complicated, outdated, and difficult to work with, making it difficult to engage in the intervention:

“*I’m used to managing a lot of things on the computer because I do accounting*,* I use the Tax Agency’s website. I’m used to seeking and finding* [information], *but I can’t do this* [referring to the Portal]. *It was awfully challenging!*” (Parent 52, mother, non-adherer, no LICBT module opened).

#### Barriers to engagement (research team)

Various external factors hindering intervention engagement were described, including the COVID-19 pandemic, which resulted in increased workload, holidays, changes in family and living situations, other illnesses in the child, and hospital check-ups:

“*Parallel to signing up for this*,* a colleague of mine that I’ve worked closely with resigned*,* and that meant an enormous amount of extra work for me* […] *therefore*,* the study fell through the cracks*,* unfortunately.*” (Parent 34, mother, non-adherer, BA).

Some parents perceived the intervention as impersonal and “template-driven”, negatively impacting engagement:

Interviewer: “*Was there anything else that you missed from the intervention and had wished for it to contain?*”

Parent: “*No*,* it was more about this individual tailoring* [of the intervention] *that is designed for every individual. But maybe it’s difficult when it’s a research project like this.*” (Parent 45, mother, non-adherer, WM).

Challenges with in-module and weekly homework exercises were described as negatively impacting engagement, including difficulties understanding the rationale for WM, the timeframe for BA, and a lack of clarity on how to submit exercises. Some parents working with BA faced challenges with overactivity rather than inactivity, finding it difficult to plan more activities. However, these difficulties were solved in collaboration with the parent guide.

For some parents, Portal usability also impacted engagement, e.g., considering the Portal to have a “boring” design, being difficult to navigate (i.e., finding completed exercises), lacking smartphone adaptation, and logging in with Bank-ID was considered complicated.

#### General experiences with participation (public contributors)

The eligibility interview and semi-structured interview before accessing EJDeR were appreciated by parents:

“*I thought they* [eligibility interview and semi-structured interview with a licensed psychologist] *worked well. They were good and pleasant conversations*,* rewarding. There was nothing strange* [with the interviews].” (Parent 35, mother, non-adherer, BA).

Contact with the parent guide via telephone was acceptable; however, those who chose video-conferencing commonly reported technical difficulties. The intervention language was acceptable, and most parents found the pace appropriate, although some perceived the tempo too intense and wished for a slower pace. Opinions on when the intervention should be offered varied and were considered individual, but generally, parents recommended offering the intervention after the child’s treatment had ended:

“*You don’t have time to reflect when you’re going in and out of the hospital*.” (Parent 44, mother, non-adherer, WM).

Parents described working with the intervention directly on the Portal, viewing the ability to print exercises as positive:

“*I did both. I did them* [exercises] *in the Portal*,* and some I printed because I thought it was better to get some kind of overview when I was writing down my activities day by day.*” (Parent 20, mother, adherer, BA).

However, external factors negatively influenced participation, such as everyday stress, life changes (e.g., changes at work, separations), and general difficulty finding time for the intervention:

“*I had the motivation*,* it’s just that I never got to it*,* and there was a lot going on at work too. So*,* I had a very big burden.*” (Participant 51, mother, non-adherer, no LICBT module opened).

The COVID-19 pandemic also impacted participation, e.g., parents became more burdened at work and tired of spending more time in front of a computer.

#### Acceptability of study procedures (research team)

Psychologist interviews before accessing EJDeR (eligibility interview and semi-structured interviews at baseline) were generally described positively:

“*Yes*,* I thought that it* [the psychologist interview] *was great. To me*,* it was all the conversations that gave me the most.*” (Parent 19, mother, adherer, BA).

However, some parents expressed discomfort and unease concerning questions about suicidality and drug use. Parents also expressed confusion concerning the rationale for interviews before accessing EJDeR, and for some parents, the support provided by psychologists after the interview was inadequate, given the sensitive information shared. One parent expressed: “*Being constantly reminded of everything… all the hard*,* and most horrible things that have happened in a life* […] *and not receiving feedback and no support around it.*” (Parent 14, mother, adherer, BA). Parents suggested a need for face-to-face interviews when sharing sensitive and potentially distressing information instead of over the telephone.

Portal assessments were perceived as too many, too repetitive, and too frequent. For some parents, they were mildly irritating; however, for others, they were an explicit barrier to intervention engagement:

“[…] *you answered the same thing over and over again. It’s the amount* [of questions] *that matters because you start to feel that… ‘yeah*,* but I’ve already answered this.”* (Parent 46, mother, non-adherer, no LICBT module opened).

Opinions on questionnaire relevancy differed. Some parents raised frustration with the perceived disconnect between the questionnaires (e.g., questions about trauma) and intervention content (e.g., lack of intervention content on trauma). Portal assessments were also perceived as valuable for self-reflection, and receiving feedback on questionnaire scores was suggested as a way to enhance their personal relevancy in the future. While reminders were generally described as helpful, they evoked feelings of stress for some parents.

#### Reminders (public contributors)

Experiences with reminders were mixed. While some parents found them helpful and necessary, others felt they were stressful and perceived them as “nagging.” However, even those who perceived the reminders as stressful acknowledged their value:

“*Yes*,* I think it was good to get the reminders. Sometimes*,* when you felt a bit stressed*,* it could be like an extra stress. But at the same time*,* if I had not received those reminders I might not have got around to doing it* [working with the intervention]. *So I think it has been good to get them.*” (Parent 2, father, adherer, BA & WM).

#### Positive experiences with participation (public contributors)

Parents described the intervention as serious and those working with it (e.g., the research team, psychologists, and parent guides) as knowledgeable. Exercises were reported to be good and relevant, and parents felt they had chosen the right module to work with. Multiple intervention components and study procedures were mentioned positively, e.g., BA, goal setting, WM, and psychologist interviews:

Interviewer: *“In your opinion*,* was behavioural activation the part that gave you the most?”*

Parent: “*Yes*,* exactly. There was a small video there with other parents that* [name of parent in case vignette] *had written a text where he described how he stopped doing things and how he lost energy.*”

Interviewer: “*Did you recognise this*?”

Parent: “*I recognised everything*,* I was almost in tears.*”

(Parent 10, father, adherer, BA).

Working with the intervention was perceived as positively impacting the family and other people surrounding the parents. Parents described exercises as effective in helping them gain insight into their feelings and plan their everyday lives.

Videos and case vignettes were appreciated. The intervention was also perceived as flexible and accessible, making it easy to participate:

“[The intervention is] *accessible timewise and accessible physically. You don’t have to go somewhere*,* and you can do it whenever you want*.” (Parent 19, mother, adherer, BA).

Weekly Portal assessments were also seen as beneficial for self-awareness, and the Portal was described as easy to use and functional. Most parents would recommend the intervention to other parents in a similar situation.

#### Appreciated parts of the intervention (research team)

The intervention was generally perceived as easy to understand and with an appropriate pace, though for some parents, the pace was too fast, and an option to pause the intervention should be provided. Overall, parents appreciated the LICBT techniques (e.g., BA, worry time, problem solving) and additional intervention content:

*“Overall*,* I thought everything was good… both keeping track of the here-and-now and my current situation*,* and working forward.*” (Parent 8, father, adherer, BA).

For those working with BA, planning and prioritising enjoyable activities, as well as gaining a better overview of their weekly activities, was described as valuable, especially for parents of young children. Parents working with WM expressed the value of differentiating between solvable and unsolvable worries and appreciated structured problem-solving. Psychoeducation was considered helpful in facilitating parents’ understanding of their current situation and difficulties:

“*The identification* [of the parent’s current situation and difficulties] *was best… I knew a bit already about what I needed to work on*,* but I got a bit more aware of that*.” (Parent 18, mother, adherer, BA).

Some parents reported relating to case vignettes, which facilitated engagement. However, for others, case vignettes were perceived as depicting parents with more severe problems and were, therefore, difficult to identify with.

#### Mechanisms of change and impact (research team)

Parents described using the intervention techniques in everyday life, e.g., setting goals, prioritising activities differently, procrastinating less, and increasing awareness of activities that helped their mood. Some parents mentioned passing on what they learned from exercises to family members, and using exercises to provide advice to others. Changing priorities resulted in increased energy, reduced depressive mood, decreased irritability and anger, and improved sleep. Parents also described “daring” to rest more and being less fearful of their worries:

“[Before EJDeR] *I’ve just removed them* [the worries]. *But then it became very clear that I have devoted myself quite a lot to cancelling and pushing away worries. So that was good*,* I think*,* that I became aware of it*,* and I practiced* [to] *actually think clearly and articulate what I was afraid of*.” (Parent 30, mother, adherer, WM).

Parents felt the intervention improved their understanding of their mood. They also reflected more on themselves and perceived their emotions as more legitimate:

“*One thing is* [gaining] *a little insight to what works. And also*,* that you can find yourself in certain behavioural patterns* […]” (Parent 36, mother, non-adherer, BA).

However, some parents reported that working with the intervention did not change anything in their everyday lives.

Negative consequences of working with the intervention were also reported, including feelings of guilt when engaging with BA, loneliness due to the online format, and difficult emotions and memories being “stirred up”.

### Intervention delivery and support

#### Opinions on internet-administered interventions (research team)

Parents described advantages to internet-administered interventions, e.g., accessibility for those living remotely, shorter waiting times, flexibility to work from different locations and at convenient times, and the ability to revisit material:

“*Yes*,* the benefit is the flexibility and so*,* and of course*,* the possibility to do it when you want to and repetition and so on. So that’s the benefit.*” (Parent 39, father, non-adherer, WM).

Disadvantages included the less personal nature of internet-administered interventions compared to face-to-face interventions, difficulty building a relationship with the parent guide, and challenges tailoring the intervention to individual needs. Parents also considered it easier to “slip away”, struggling to allocate time, e.g., taking time off work, and being more likely to withhold the truth when talking to a parent guide. Internet-administered interventions were considered to require self-discipline and some technical knowledge:

“*The drawback is*,* as mentioned*,* if you’re not very technically skilled*,* paired with the navigation* […] *in the Portal may not be the easiest. And you actually have to do things yourself*,* so if you don’t* [do it] *it won’t happen. That’s the biggest drawback.*” (Parent 1, father, adherer, BA & WM).

However, internet-administered interventions were viewed as an acceptable substitute for those voicing a preference for face-to-face interventions.

#### Parent guide (public contributors)

Parents perceived the parent guide as supportive, helpful, and validating their thoughts and emotions:

“*Yes*,* I think it* [communication with parent guide] *was very good. She was always quick to reply when I wrote something and* [I had] *very good conversations with her*.” (Parent 35, mother, non-adherer, BA).

Receiving written feedback was generally perceived as a positive and professional experience, especially when compared to verbal feedback alone:

“*I think it was good to get this weekly* [feedback], *to get confirmation on what you had done. You see that you’re heading in the right direction and you get some validation of your* [own] *thoughts and emotions*.” (Parent 15, mother, adherer, BA).

#### Communication and support (research team)

The parent guide was generally appreciated and described positively, e.g., being easy to contact, encouraging, genuine, responsive, understanding, and warm. Parents valued receiving both general encouragement and intervention-specific information from parent guides. The initial assessment session (telephone or video-conferencing) was important for building a relationship with the parent guide, with parents wishing for more contact with their parent guide. However, combining guidance via telephone or video conference and receiving written feedback on exercises was considered beneficial:

“*Well*,* I think that* [contact with the parent guide] *was great. You felt that you could always ask questions if you didn’t figure it out right away and got good support with the program itself… I had a little difficulty with certain parts*,* so I thought that was great!*” (Parent 35, mother, adherer, BA).

Those parents who chose video-conferencing described encountering technical difficulties. Technical challenges were also described with written communication on the Portal, e.g., difficulties finding parent guide feedback and not being able to respond to messages via mobile phones:

“*It was a bit complicated. You couldn’t reply to the SMS* [on the phone], *you had to login* [to the Portal] *and write there so that she* [the parent guide] *got the message*,* and then she messaged me through SMS*.” (Parent 2, father, adherer, BA &WM).

A few parents considered their parent guide as too “perky” and overly validating, and wished for more of a “push”.

### Areas for improvement

#### Wishes, opinions, and suggestions for improvements (public contributors)

A need for additional support was expressed, such as more interaction with psychologists and conversations with other parents in a similar situation. Parents also wished for individual tailoring of the intervention pace and frequency of contact with parent guides. Compatibility with smartphones and tablets was identified as another area for improvement:

“*I had probably been more active and maybe done these exercises better had it been possible to fill them out on the cell phone*.” (Parent 19, mother, adherer, BA).

Furthermore, parents suggested making the intervention easier to navigate, such as helping them find previously set goals and including a timeline illustrating the intervention’s progression. They further recommended reducing the number of questionnaires and ensuring consistent scales across them:

“*Easier setup*,* not that many questions* [parent referring to questionnaires]." (Parent 52, mother, non-adherer, no LICBT module opened).

#### Suggestions and improvements (research team)

Some parents described the intervention as too generic and not sufficiently tailored to the experience of being a parent of a child treated for cancer, nor to individual needs.

“*I think it would have been good to spend some more time in the beginning on tailoring the content*,* so to say*.” (Parent 7, father, adherer, BA).

Parents suggested acknowledging trauma and emotions related to the treatment period more. Including a family and parenting perspective, addressing changing family dynamics and relationships outside the family was recommended.

Suggestions for improvements included more video material, the ability to respond to messages via SMS, access to all intervention material, and contact with other parents. Parents who did not adhere struggled to identify specific changes that would have facilitated adherence, though suggested doing exercises together with the parent guide, more individual tailoring, and fewer questionnaires.

## Discussion

### Main findings

We explored the acceptability and feasibility of EJDeR and ENGAGE study procedures from the perspective of parents to inform future modifications and refinements to the intervention and study procedures before progression to the CHANGE-pilot trial. Two separate data analyses were conducted, one by the public contribution group and one by research team members, with overall findings suggesting that EJDeR and study procedures are acceptable and feasible.

Overall, parents considered EJDeR to be acceptable and relevant, and parents expressed positive attitudes towards internet-administered support e.g., being accessible, convenient, and flexible, a finding consistent with other literature [16]. However, for some, EJDeR failed to meet their needs, or parents perceived themselves as feeling too well to benefit from the intervention. A potential reason for this may relate to parents not needing to reach clinical cut-offs for symptoms of depression and/or anxiety to participate, but rather, we targeted parents of children treated for cancer with a self-reported need for psychological support. Additionally, some parents misunderstood the nature of the study and joined for altruistic reasons, e.g., expecting to contribute to intervention development to help others rather than receiving support themselves. Altruism is a commonly reported reason for research participation [51]. However, altruism may also be linked to perceived personal benefit and risk [52]. Given the time and commitment needed to participate in the intervention and study, coupled with some parents not perceiving an intervention need, the perceived personal benefit of participation may have been low for some parents joining the study for altruistic reasons, potentially contributing to low intervention adherence and/or study retention.

A related point concerning study understanding was raised and discussed by public contributors during data analysis workshops. Public contributors reflected that the general public might associate the word “study” with survey research or providing feedback, rather than intervention research. The finding that some parents did not understand they would receive an intervention underscores the importance of providing clear study information [53]. To improve study understanding in the future (i.e., in the CHANGE-pilot trial), we plan to hold extended discussions with study participants [54] and user-test participant-facing study information [55], i.e., co-designing study information sheets with public contributors to develop more comprehensible and understandable study materials [56].

Parents asked for greater focus on the wider family to further improve intervention acceptability. Interviews prior to accessing EJDeR suggested parents experience concerns regarding family relationships (e.g., feeling misunderstood by relatives and friends) and parenting a child treated for cancer (e.g., overprotecting the child) [28]. Other research indicates that some parents of children treated for cancer experience difficulties with relationships within their family [57], and remain vigilant about potential illness symptoms after end of treatment [58]. Although a Cochrane review found insufficient evidence for the effect of various psychological interventions on family functioning [59], a recent RCT suggests a family-focused CBT intervention may improve depression and PTSS in parents of children treated for cancer [60]. Whilst the effectiveness of family-focused interventions for the population remains unclear, our findings suggest a need for psychological interventions to recognise the parent within a broader family context.

Some parents also expressed wanting an increased focus on trauma and the cancer experience, which aligns with our previous research showing clinically relevant levels of PTSS among parents years after their child’s treatment [61]. E-therapists guiding EJDeR also mentioned, when interviewed, that some parents experienced psychological difficulties, such as PTSS, that were not targeted by EJDeR [62]. At the time EJDeR was developed, there was limited evidence supporting LICBT and iCBT for PTSD [63–65]. However, the evidence base for iCBT for PTSD has since grown [66]. Findings, therefore, suggest a need for clinical modifications to EJDeR to include psychoeducation and techniques to manage PTSS.

Barriers to engagement included study procedures, such as the number and frequency of Portal assessments and technical difficulties on the Portal. E-therapists also perceived Portal assessments as stressful for parents, negatively impacting intervention engagement and study retention [62]. Given extensive questionnaires may lead to a loss of follow-up and reduced statistical power [67] there is a need to reduce the amount and frequency of questionnaires to improve retention. Parents suggested receiving feedback on questionnaire responses may also improve engagement, a finding reported in other trials of internet-administered psychological interventions [68]. Provision of feedback has been found to overcome challenges with questionnaires being perceived as too symptom-focused and failing to capture the complexity of experiences [69]. E-therapists using questionnaire responses for clinical decision-making may also represent a way to increase intervention relevancy, enhance engagement, and increase retention [70].

Some parents perceived a lack of Portal usability, especially concerning smartphone and tablet compatibility. Other technical challenges were experienced navigating the Portal, especially concerning written communication on the Portal, e.g., difficulties finding e-therapist feedback. User-friendly, well-organised, clear designs are critical when implementing iCBT interventions [71, 72]. Involving end-users in co-designing solutions for technical challenges may be necessary before the CHANGE-pilot trial, given iCBT usability problems are common [73]. Technical difficulties with iCBT interventions are also associated with reduced retention rates [74], further emphasising the need to improve intervention usability.

While parents recognised the benefits of an internet-administered intervention, some found the intervention impersonal and wished for more e-therapist contact. This finding aligns with previous research suggesting internet-administered interventions may be perceived as less personal than face-to-face treatments [75], which are generally preferred [76]. However, guided iCBT interventions for psychiatric and somatic disorders in adults show overall equivalent effect sizes as face-to-face therapy [77], and a high level of patient-therapist alliance is reported in iCBT interventions for depression and anxiety disorders in adults [78, 79]. Guided iCBT has been found to be associated with higher ratings of patient-therapist alliance than unguided for mild to moderate depression [80], and guidance considered crucial for iCBT engagement [72, 81]. Future clinical modifications to EJDeR may include the provision of more human interaction and synchronous communication (i.e., via telephone or video-conferencing), as also suggested by e-therapists [62] to improve intervention engagement and adherence.

### Public contribution

This study aimed to involve public contributors in all data analysis steps, including dissemination. Whilst involving public contribution in qualitative data analysis is becoming more common, some examples do not include public contributors in all data analysis steps, e.g., analysing pre-selected quotations rather than an entire data set [82]. In other examples, analysis is conducted by a mixed group of researchers and people with lived experience [82, 83], which may result in the voices of people with lived experience being lost [82] and a lack of easy separation between researcher and public contributor analysis [84]. To overcome these limitations, we conducted two separate data analyses, one by the public contribution group and one by research team members, until final report writing, which was led by the research team but informed by memos written by each analysis team and reviewed by public contributors. Whilst this approach facilitated easy separation of the analysis and minimised losing the voice of public contributors, it resulted in two often repetitive and overlapping data analyses. Alternatively, a “whole-team” approach could have been adopted whereby initial data analysis steps are conducted separately, followed by “whole-team” meetings to co-develop the analytical framework coupled with continuous reflection on how to ensure the voices of public contributors are not lost, especially during the writing phase [50].

Overall, the public contributors’ analysis resulted in findings similar to those of the research team, strengthening the credibility of the findings. For example, the public contributors’ categories “Expectations/information beforehand”, “Parent Guide”, and “Wishes, opinions, and suggestions for improvement” are close to the research team’s categories “Before the study”, “Communication and support”, and “Suggestions and improvements”, respectively. However, there were also important differences between the analyses. Public contributors highlighted nuances overlooked by the research team, informing important future modifications and refinements to EJDeR and study procedures. One notable insight concerned reflections from public contributors on parents’ potential interpretation of the word " study”, e.g., associations with survey participation (i.e., answering questions) versus receiving an intervention, which may have impacted study and intervention expectations. Public contributors also reflected that parents tended to perceive EJDeR and ENGAGE study procedures as an “entire package”, making distinguishing between intervention versus study procedure acceptability and feasibility challenging. This is reflected in the analyses. For example, the research team’s category “Acceptability of study procedures” focused on data relating to study procedures, e.g., interviews with psychologists, Portal assessments (frequency and relevancy), and reminders. Data concerning the acceptability of EJDeR was categorised by the research team under separate categories such as “Appreciated parts of the intervention”, “Communication and Support”, and “Barriers to engagement”. Conversely, public contributors commonly combined data reflecting both EJDeR and study procedures, e.g., categories for “General experiences of participation” and “Positive experiences with participation”, with the exception of the category “Reminders”, potentially pointing to reminders being more clearly separated from the intervention than other study procedures from the parents’ perspective. This difference in understanding of concepts related to the intervention versus study procedures is an important finding as it has implications for future communication with study participants (e.g., the potential need for clarifications in study information and modifications of interview questions to explore acceptability and feasibility). However, this difference in understanding presented some challenges for report writing, with categories presented under key areas sometimes overlapping or lacking in nuance. For example, the research team presented parents’ opinions on when EJDeR should be offered alongside parents’ general experiences of availability of support in the category “Need for support”, presented in the key area “Expectations and needs”. Public contributors presented similar data in the category “General experiences with participation”, presented in the key area “Barriers, facilitators and experiences with participation”. Another example is that the research team presented both technical and relational aspects concerning communication with the parent guide in the “Communication and support” category, presented in the key area “Delivery and support”. Public contributors categorised technical aspects under “General experiences with participation”, presented in the key area “Barriers, facilitators and experiences with participation”, and relational aspects under “Parent guide”, presented in the key area “Delivery and support”. Again, adopting a “whole-team” approach could have been beneficial for structuring the results [50].

Further, the research team identified more academic themes, i.e., “Mechanisms of change and impact”, informed by the language used in process evaluations of complex interventions [85]. For public contributors, intervention impacts were captured under “Positive experiences with participation”. Public contributors also placed more emphasis on parents’ lack of time as a stress factor impacting engagement and a need for future research to clarify better the time commitment required for participation. Findings suggest public contributors are more likely to interpret the data through the lens of their own personal experience and other research has found public contributors place more emphasis on data relating to experiences and emotions [86]. During analysis workshops, public contributors reflected that their own lived experience as parents of a child treated for cancer facilitated their more nuanced interpretation of results. The benefits of this insider perspective have been highlighted in other research, emphasising the importance of involving public contributors to facilitate the development of a more comprehensive and nuanced analysis [87].

### Implications and future directions

To enhance the acceptability and feasibility of EJDeR and study procedures, findings will be used to inform modifications and refinements to EJDeR and study procedures for the CHANGE-pilot trial. These modifications and refinements are provided in Table 4.

**Table 4:** Future modifications and refinements to EJDeR and study procedures

Findings	Suggested modifications and refinements
Acceptability and feasibility of EJDeR	
Module content: A need for more focus on the cancer experience.	Intervention content further tailored to the population, e.g., the experience of being a parent of a child treated for cancer by including more cancer-related examples throughout the modules.
Appreciation of video content.	Addition of two video case vignettes, focusing on recovery
Psychoeducation: A need for more focus on PTSS, relationships, and the wider family.	Provision of psychoeducation and techniques to manage PTSS. Provision of psychoeducation on relationships and parenting difficulties commonly experienced by parents of children treated for cancer.
Portal: The Portal lacked user-friendliness.	Development of a more user-friendly version of the Portal. Involvement public contributors in designing and testing the new Portal.
Communication and support: More contact with the e-therapist wanted.	Provision of an additional follow-up support session via telephone or video-conferencing to parents.
Acceptability and feasibility of study procedures	
Study information: Some parents did not fully understand that the study contained a self-help intervention.	Provision of extended discussions with potential participants before conducting eligibility interviews to ensure understanding of study information. Involvement of public contributors in co-designing study information.
Questionnaires: Questionnaires were reported as too many and frequent.	Reduction of the number and frequency of questionnaires.

### Strengths and limitations

The study has some limitations. First, the large number of interviews analysed may not have been necessary, complicating data management and potentially the depth and nuance of the analysis [88]. Second, interviews were mainly conducted by external licensed psychologists who were not trained in qualitative interviewing. Paired with the relatively detailed and focused interview guide, this may have resulted in explorative follow-up questions not always being asked, resulting in a lack of in-depth information. Third, findings might not be transferable to other populations, given our sample characteristics. Specifically, parents were predominantly female, highly educated, and had low levels of depression and anxiety. Additionally, a higher proportion of parents adhering to EJDeR participated in the post-treatment (12 weeks) interviews (63.5%) compared to the overall ENGAGE sample (47.9%), potentially missing reasons for low acceptability and adherence.

Despite these limitations, the study has several strengths. First, all parents remaining in ENGAGE at post-treatment (12 weeks) were contacted for the post-treatment interview, ensuring a sample that included both adherers and non-adherers, providing a comprehensive understanding of the acceptability and relevance of the intervention and study. Second, the sample size of 52 interviews can be considered large [89], capturing a wide range of experiences and opinions. Third, involving public contributors in conducting data analysis separate from the research team is a novel approach [34], yielding and contributing important insights and findings [50].

## Conclusions

This study aimed to explore the acceptability and feasibility of EJDeR and ENGAGE study procedures from the perspective of parents to inform future modifications and refinements to the intervention and study procedures before progression to the CHANGE-pilot trial. Overall, EJDeR and ENGAGE study procedures were found to be acceptable and feasible, but some modifications and refinements were suggested. A broader focus of EJDeR with additional material on PTSS, relationships, and parenting will be adopted. The Portal will be developed to be more user-friendly, and a follow-up support session with an e-therapist will be added. Regarding study procedures, study information will be clarified and enhanced, and the number of questionnaires will be reduced. Adopting a novel approach to qualitative analysis by involving public contributors separately in each data analysis step resulted in a more nuanced understanding of the data.

## Electronic supplementary material

Below is the link to the electronic supplementary material.

Supplementary Material 1

Supplementary Material 2

Supplementary Material 3

## Data Availability

Due to the nature of this research, participants did not agree to share their data publicly, so supporting data is not available, and further ethical approval would be needed to share this data.

## Abbreviations

BA: Behavioural Activation
CBT: Cognitive Behavioural Therapy
GAD: Generalised Anxiety Disorder
GAD-7: Generalised Anxiety Disorder 7-item scale
iCBT: Internet-Administered Cognitive Behavioural Therapy
IPE: Introduction and Psychoeducation
LICBT: Low-intensity Cognitive Behavioural Therapy
M.I.N.I.: Mini-International Neuropsychiatric Interview
MRC: Medical Research Council
PCL-5: Post-Traumatic Stress Disorder Checklist for DSM-5
PCL-C: Post-Traumatic Stress Disorder Checklist-Civilian
PHQ-9: Patient Health Questionnaire 9-item scale
PTSS: Post-Traumatic Stress Symptoms
RCT: Randomised Controlled Trial
RP: Relapse Prevention
SMS: Short Message Service
UC: Usual Care
WM: Worry Management
