# Supplementary material for: The acceptability and feasibility of an internet-administered, guided, low-intensity cognitive behavioural therapy intervention for parents of children treated for cancer: findings from a qualitative study involving public contributors
Source: BMC Psychiatry. 2025 May 16;25:499. doi: 10.1186/s12888-025-06897-y (PMC12084959; doi:10.1186/s12888-025-06897-y)
Supplement: Supplementary file 2 — Supplementary Material 2 [file 12888_2025_6897_MOESM2_ESM.docx]

**Additional File 2**. Sociodemographic and clinical characteristics for parents and children

| **Sociodemographic and clinical characteristics** | | | |
| --- | --- | --- | --- |
| **Parent self-report data** | **Total (N=52)**  **n (%)** | **Fathers (N=17)**  **n (%)** | **Mothers (N=35)**  **n (%)** |
| Relationship to child treated for cancer |  |  |  |
| Fathers | 17 (32.7) | 17 (100) | 0 (0.0) |
| Mothers | 35 (67.3) | 0 (0.0) | 35 (100) |
| Age (years) mean (SD) range | 41.7 (5.6) 32-59 | 43.8 (4.8) 36-57 | 40.7 (5.0) 32-59 |
| Highest level of education |  |  |  |
| Secondary (Upper and Lower) | 7 (13.5) | 2 (11.8) | 5 (14.3) |
| Post-secondary non-tertiary | 2 (3.8) | 0 (0.0) | 2 (5.7) |
| Tertiary | 41 (78.8) | 13 (76.5) | 28 (80.0) |
| PhD | 2 (3.8) | 2 (11.8) | 0 (0.0) |
| Housing situation |  |  |  |
| Rental | 2 (3.8) | 0 (0.0) | 2 (5.7) |
| Apartment ownership | 12 (23.1) | 4 (23.5) | 8 (22.9) |
| House ownership | 37 (71.2) | 12 (70.6) | 25 (71.4) |
| Other | 1 (1.9) | 1 (5.9) | 0 (0.0) |
| Region of birth |  |  |  |
| Nordic countries^a^ | 46 (80.8) | 15 (88.2) | 31 (88.6) |
| Asia | 3 (5.8) | 1 (5.9) | 2 (5.7) |
| Europe (excluding Nordic countries) | 3 (5.8) | 1 (5.9) | 2 (5.7) |
| Employment status |  |  |  |
| Employed | 47 (90.4) | 17 (100) | 30 (85.7) |
| Unemployed | 5 (9.6) | 0 (0.0) | 5 (14.3) |
| Previous psychological treatment |  |  |  |
| Yes | 27 (51.9) | 6 (36.3) | 21 (60.0) |
| No | 25 (48.1) | 11 (64.7) | 14 (40.0) |
| Relationship status |  |  |  |
| Partner | 43 (82.7) | 13 (76.5) | 30 (85.7) |
| Single | 9 (17.3) | 4 (23.5) | 5 (14.3) |
| Physical health problems |  |  |  |
| Yes | 16 (30.8) | 5 (29.4) | 11 (31.4) |
| No | 36 (69.2) | 12 (70.6) | 24 (68.7) |
| Previous traumatic/difficult life event |  |  |  |
| Yes | 45 (86.5) | 14 (82.4) | 31 (88.6) |
| No | 7 (13.5) | 3 (17.6) | 4 (11.4) |
| Age of child (years) mean (SD) range^b^ | 10.1 (4.7) 2-24 | 11.0 (4.7) 6-24 | 9.7 (4.8) 2-21 |
| Sex of child^b^ |  |  |  |
| Female | 23 (47.9) | 7 (50.0) | 16 (47.1) |
| Male | 25 (52.1) | 7 (50.0) | 18 (52.9) |
| Cancer diagnosis^b^ |  |  |  |
| Leukemia | 25 (52.1) | 9 (64.3) | 16 (47.1) |
| Blastoma | 10 (20.8) | 3 (21.4) | 7 (20.6) |
| Lymphoma | 7 (14.6) | 2 (14.3) | 5 (14.7) |
| CNS tumor | 4 (8.3) | 0 (0.0) | 4 (11.8) |
| Germ cell tumor | 1 (2.1) | 0 (0.0) | 1 (2.9) |
| Sarcoma | 1 (2.1) | 0 (0.0) | 1 (2.9) |
| **Swedish Childhood Cancer Registry data^b,c^** | **Total (N=45)**  **n (%)** | **Fathers (N=13)**  **n (%)** | **Mothers (N=32)**  **n (%)** |
| Age of child (years) mean (SD) range | 10.1 (4.7) 4-24 | 11.1 (4.9) 6-24 | 9.7 (4.6) 4-21 |
| Sex of child |  |  |  |
| Female | 23 (51.1) | 7 (53.8) | 16 (50.0) |
| Male | 22 (48.9) | 6 (46.2) | 16 (50.0) |
| Cancer diagnosis |  |  |  |
| Leukemia | 24 (53.3) | 8 (61.5) | 16 (50.0) |
| Blastoma | 10 (22.2) | 3 (23.1) | 7 (21.9) |
| Lymphoma | 7 (15.6) | 2 (15.4) | 5 (15.6) |
| CNS tumor | 4 (8.9) | 0 (0.0) | 4 (12.5) |

*Note*. Both data from the Swedish Childhood Cancer Registry and from parents (self-report) was collected as the Swedish Childhood Cancer Registry is not updated in real-time and therefore the data provided by the Swedish Childhood Cancer Registry may not have been completely up-to-date at the time participants were recruited into the study.

^a^Including: Denmark, Finland, Norway, and Sweden

^b^Data for children with both parents participating in the study, out of the whole study sample (n = 4), are only included once.

^c^Data for n = 3 children treated for cancer whose parents were recruited via online advertisements via cancer organisations and interest groups was not available from the Swedish Childhood Cancer Registry. Data for children with both parents participating in the study (n = 3) are only included once.
