# Supplementary material for: The acceptability and feasibility of an internet-administered, guided, low-intensity cognitive behavioural therapy intervention for parents of children treated for cancer: findings from a qualitative study involving public contributors
Source: BMC Psychiatry. 2025 May 16;25:499. doi: 10.1186/s12888-025-06897-y (PMC12084959; doi:10.1186/s12888-025-06897-y)
Supplement: Supplementary file 3 — Supplementary Material 3 [file 12888_2025_6897_MOESM3_ESM.docx]

Additional File 3. Semi-structured interview guide

General instructions before the interview:

- Make sure that the telephone is charged and works
- Have paper and pencil to hand to take notes
- Make sure that the recorder is turned on
- Ask for consent for the interview to be recorded. If the participant does not consent the interview cannot be conducted, but the participant can remain in the project
- Thank the participant for agreeing to take part
- State that the aim of the interview is that we wish to know how they experienced working with the program
- FOR THOSE WHO DID NOT FINISH THE PROGRAM: State that you know that they did not finish the program, and that it is important for our research to understand why
- State that the interview will last 20-40 minutes
- Encourage the participant to answer in their own words, at their own pace
- State that the participant can change their responses, would they change their mind during the interview
- State that the participant can stop the interview, should they wish to
- State that everything that is said will be treated confidentially

NOTE: ENCOURAGE THE PARTICIPANT TO ELABORATE THEIR RESPONSES AND ASK FOLLOW-UP QUESTIONS!

| Topic | Questions |
| --- | --- |
| 1. General | 1. Did the project and the program content match the information you received in the invitation letter or on social media?  2. How did you experience the interview process before gaining access to the program? |
| 2. Exercises | 1. How did you complete the exercises?   - Via the Portal - On paper (printed exercises) - With other tools you usually use, e.g., apps on your telephone, your diary - In your everyday life   2. Were some exercise(s) extra valuable?  3. Were some exercise(s) less valuable?  4. Were some exercise(s) difficult to understand?  5. How relevant were the exercise(s) for your situation?  6. Have some of the exercise(s) changed any of your behaviour(s)?  7. Have you used some of the exercise(s) in your everyday life? |
| 3. Parent guide | 1. How did you experience the contact with your parent guide?  2. Did you keep in touch with your parent guide via telephone or video-conferencing? Why did you choose this communication form? How did you experience communicating with your parent guide that way?  3. How did you experience the written feedback from your parent guide? If not mentioned by parent: ask about frequency, content, and technique. |
| 4. Portal | 1. How did you experience the Portal’s visual design?  2. How did you experience using the functions on the Portal? |
| 5. Questionnaires and reminders | 1. How did you experience answering questions on the Portal before you started working with EJDeR?  2. How did you experience answering questions on the Portal every week?  3. How did you experience receiving reminders via /e-mail/telephone about answering questions on the Portal? If not mentioned by parent: ask about frequency, content, and technique.  4. How do you experience answering questions on the Portal now when you have finished working with EJDeR?  5. Is there anything that has affected how you experience answering questions on the Portal? If so, what? |
| 6. For those who did not complete the program | 1. Why did you not complete your work with the program?  2. Is there anything we could have done to help you complete your work with the program? |
| 7. Concluding questions | 1. What expectations did you have of the program?  2. Did the content of the program meet expectations? If not, why?  3. Did you miss something in the program?  4. What part of the program was most important to you?  5. Could we improve the program? If not mentioned by parent: ask about content, exercises, communication with the parent guide (video-conferencing, telephone, e-mail, SMS), pace, language. 6. Would you recommend the program to someone in a similar situation as yours? If yes: why? If no: why not?  7. How long after a child’s treatment ends do you think the program should be offered to parents?  8. What are the advantages and disadvantages with internet-administered support compared to face-to-face meetings?  9. Is it okay if I contact you again in the future if I would like to ask any additional questions or for clarification? |

Note. When talking about the program we refer to the intervention [Swedish acronym for int**E**rnetbaserat s**J**älvhjälpsprogram för föräl**D**rar till barn som avslutat en behandling mot canc**eR**]
